# Zugangswege und Fixation kindlicher Schenkelhalsfrakturen – transglutealer Zugang

**DOI:** 10.1007/s00064-020-00694-4

**Published:** 2021-01-21

**Authors:** Kai Ziebarth, Nadine Kaiser, Theddy Slongo

**Affiliations:** Abteilung Kinderorthopädie, Kinderchirurgische Universitätsklinik, Inselspital Bern, Freiburgstr., 3010 Bern, Schweiz

**Keywords:** Plattenosteosynthese, Abduktoren, Femurkopfnekrose, Stabilisierung, Reposition, Plate osteosynthesis, Hip abductor, Avascular necrosis, Stabilization, Fracture reduction

## Abstract

**Operationsziel:**

Die Hüftkopfzirkulation schonende, anatomische Reposition und sichere Stabilisierung von Schenkelhalsfrakturen im Kindesalter über einen transglutealen Zugang.

**Indikationen:**

Intra-extraartikuläre proximale Femurfrakturen (Schenkelhalsfrakturen) AO 31-M/2.1 I‑III; 31-M/3.1 I‑III; 31-M/3.2 I‑III.

**Kontraindikationen:**

Keine.

**Operationstechnik:**

Präparation eines Muskellappens durch Ablösen des proximalen M. vastus lateralis inklusive des anterolateralen Anteils des M. glutaeus medius vom proximalen Femur respektive Trochanter major. Ablösen des glutaeus minimus von der Gelenkkapsel und Weghalten nach dorsal, ohne die Insertion des Muskels vollständig abzulösen. Die anterolaterale Gelenkkapsel kann nun vollständig exponiert werden. Arthrotomie der Gelenkkapsel und Darstellen des Schenkelhalses. Unter Sicht nun vorsichtige, kontrollierte Reposition der Fraktur unter Schutz der retinakulären Gefäße.

**Weiterbehandlung:**

Mobilisation an Gehstöcken. Abstellen des Fußes erlaubt. Zur vollständigen Anheilung der Hüftabduktoren sollte eine aktive Abduktion sowie passive Adduktion für 4 bis 6 Wochen (je nach Alter des Patienten) vermieden werden.

**Ergebnisse:**

In der eigenen Klink zeigten sich in den letzten 10 Jahren exzellente Ergebnisse bei 29 Patienten nach Behandlung von kindlichen Schenkelhalsfrakturen mit diesem Operationszugang. Eine operationsbedingte Femurkopfnekrose trat nicht auf.

## Abstract (en)

**Objective:**

Transgluteal approach for anatomical reduction of femoral neck fractures (extra-intraarticular) in children under preservation of the blood supply of the femoral head.

**Indications:**

Femoral neck fractures AO 31-M/2.1 I‑III; 31-M/3.1 I‑III; 31-M/3.2 I‑III.

**Contraindications:**

None.

**Surgical technique:**

Preparation of a muscular flap including the proximal insertion of the vastus lateralis muscle and approximately one third of the gluteus medius muscle. Elevation of the gluteus minimus muscle from the hip capsule without completely detaching it from its insertion. Exposure of the anterolateral hip capsule and capsulotomy followed by controlled reduction of the fracture fragments without compromising the retinacular vessels.

**Postoperative management:**

Touch-down weightbearing for 4–6 weeks (age dependent). To protect the healing of the abductors, active abduction or passive adduction prohibited for 4–6 weeks. Consolidation radiographs 4–6 weeks postoperatively.

**Results:**

Excellent results in 29 patients subsequently treated in the last 10 years by the transgluteal approach. No cases of avascular necrosis of the femoral head by this procedure.

## Vorbemerkungen

Schenkelhalsfrakturen im Kindesalter weisen eine niedrige Inzidenz von 1 bis 2 Frakturen pro Jahr auf, was sich auch in etwa mit den persönlichen Erfahrungen der Autoren über die letzten Jahre deckt [1, 2]. Das bedeutet, dass Schenkelhalsfrakturen in etwa 0,3–0,5 % aller Frakturen bei Kindern vorkommen. Trotz oder gerade wegen des relativ seltenen Auftretens dieser Frakturen ist die Durchführung einer korrekten Behandlung umso wichtiger, da die insuffiziente Behandlung mit konsekutiver Fehlstellung, Hüftsteife bis hin zur Notwendigkeit einer Hüftprothese für den jungen Patienten schwerwiegende Folgen im weiteren Leben hinsichtlich sozialer Integrität (Sport, Freizeit) oder auch Berufswahl haben kann. Um eine möglichst komplikationsarme Behandlung zu ermöglichen, sollte die operative Reposition von Schenkelhalsfrakturen beim Kind analog zur Behandlung des Erwachsenen so schnell wie möglich erfolgen [3]. Die am meisten gefürchtete Komplikation ist die Femurkopfnekrose durch eine Schädigung der Hüftkopfdurchblutung durch das Trauma selbst oder durch eine unkontrollierte, meist geschlossene Reposition oder aber auch durch eine Überreposition mit sekundärer Schädigung der hüftkopfversorgenden Gefäße [4]. Die Behandlung von Schenkelhalsfrakturen im Kindesalter mittels transglutealen Zugangs gewährt eine exzellente Übersicht, sodass eine anatomische Reposition der Fraktur unter Erhaltung der Femurkopfdurchblutung möglich ist. Die Fixation der Fraktur kann alternativ mittels Schrauben, Drähten oder einer Platte erfolgen. Schenkelhalsfrakturen bei Kindern können ebenfalls durch die AO (Arbeitsgemeinschaft für Osteosynthesefragen) Pediatric Comprehensive Classification of Long-Bone Fractures (PCCF) klassifiziert werden [5].

## Operationsprinzip und -ziel

Exposition des Oberschenkelhalses durch Präparation eines Muskellappens/Sehnenlappens bestehend aus dem proximalen Anteil des M. vastus lateralis inklusive des anterolateralen Anteils des M. glutaeus medius. Ablösen des M. glutaeus minimus von der Gelenkkapsel und Weghalten mittels eines tiefen, gerundeten Hakens nach dorsal, ohne die Insertion des Muskels am Trochanter major dabei vollständig abzutrennen. Die anteriore sowie inferiore (entlang des Kalkars) Gelenkkapsel kann nun vollständig exponiert werden. Bei intraartikulären Frakturen T‑förmige Arthrotomie der Gelenkkapsel und Darstellen des Schenkelhalses respektive der Fraktur. Unter Sicht kann nun eine vorsichtige, kontrollierte Reposition der Fraktur unter Schutz der retinakulären Gefäße erfolgen. Das Grundprinzip besteht in der Simulation eines Trochanter-Flip-Zuganges, ohne den Trochanter zu osteotomieren. Dies sollte absolut vermieden werden, da sonst die Stabilität der Plattenosteosynthese gefährdet wird.

## Vorteile

- Darstellung des Schenkelhalses/Hüftgelenkes bei intraartikulär gelegenen Frakturen
- Exzellente Übersicht der Fraktur, besonders im Kalkarbereich
- Anatomische Reposition unter Visualisierung der Fraktur
- Schonung der hüftkopfversorgenden Blutgefäße durch Vermeidung einer Überreposition oder unkontrollierten Manipulation
- Visuelle Kontrolle der Hüftkopfzirkulation durch Anbohren mittels 1,0-mm-Bohrers

## Nachteile

- Aufwendiger Zugang mit relativ großer Operationsnarbe
- Ablösen von Muskeln mit der Gefahr des Kraftverlustes
- Eröffnen des Gelenkes bei intraartikulären Frakturen mit Gefahr von intraartikulären Verwachsungen
- Erfordert größere Erfahrung in der Hüftchirurgie und exakte Kenntnisse der Anatomie

## Indikationen

- Dislozierte Schenkelhalsfrakturen AO 31-M/2.1I-III; 31-M/3.1 I‑III; 31-M/3.2 I‑III [4]
- Intertrochantäre Frakturen

## Kontraindikationen

- Keine

## Patientenaufklärung

- Allgemeine Operationsrisiken: Infektion, Blutung, Gefäß‑/Nervenverletzungen, Narkoserisiko
- Operationsnarbe ca. 10–15 cm
- Risiko der Muskelschwächung durch Ablösen von M. vastus lateralis und M. Glutaeus medius
- Schädigung (Traktion) des N. glutaeus superior (durch Traktion bei Weghalten des M. Glutaeus medius und minimus nach dorsal)
- Repositionsverlust
- Malunion
- Nonunion
- 4 bis 6 Wochen Mobilisation an Gehstöcken (Touch-down-Belastung)

## Operationsvorbereitungen

- Analyse der radiologischen Diagnostik (Frakturmuster)
- Auswahl des Osteosynthesematerials (von den Autoren wird eine Plattenosteosynthese, wie in der Operationstechnik beschrieben, empfohlen)
- Selten CT(Computertomographie)- oder MRT(Magnetresonanztomographie)-Diagnostik notwendig (z. B. Luxationsfrakturen, zusätzlich Acetabulumfraktur)
- Exakte Kenntnis der proximalen femoralen Anatomie

## Instrumentarium

- Hohmann-Haken (8 + 16 mm)
- An der Spitze abgerundete Hohmann-Haken (sog. weiche Eva-Haken)
- Langenbeck-Haken
- Vollgewinde-Kirschner-Drähte/Kirschner-Drähte (Joystick, präliminare Fixation)
- Plattenosteosynthese (z. B. LCP Pediatric Hip Plate, Depuy Synthes, Zuchwil, Schweiz)
- Kanülierte Schrauben (eher nicht empfohlen)
- Bildwandler

## Anästhesie und Lagerung

**Figure 1:**
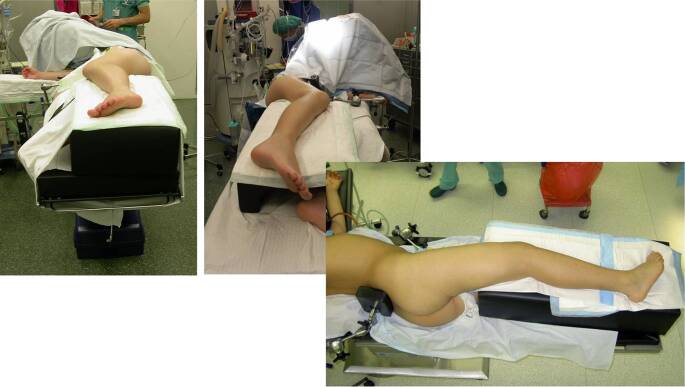

- Intubationsnarkose inklusive vollständiger Muskelrelaxation des Patienten bis zur definitiven Fixation der Fraktur
- Seitenlagerung (v. a. bei adipösen Patienten empfohlen, bessere Übersicht) mit stabiler Auflage des zu operierenden Beines, vorzugshalber stabiler Lagerungsblock (Abb. 1)
- Rückenlagerung möglich (von den Autoren nicht empfohlen)
- Gewichtsadaptierte Antibiotikaprophylaxe

## Operationstechnik

Abb. 2, 3, 4, 5, 6, 7, 8, 9, 10, 11, 12

**Figure 2:**
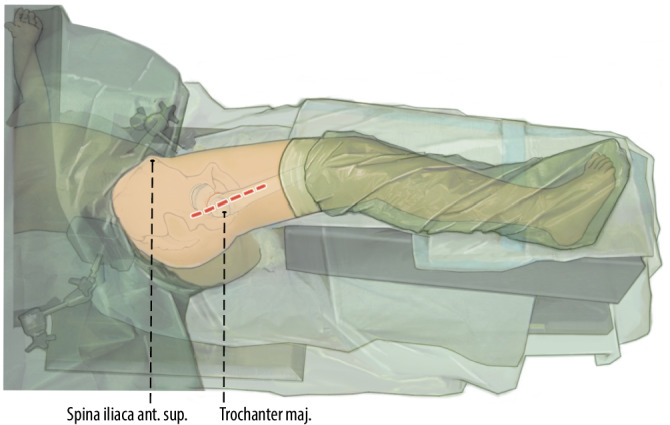

**Figure 3:**
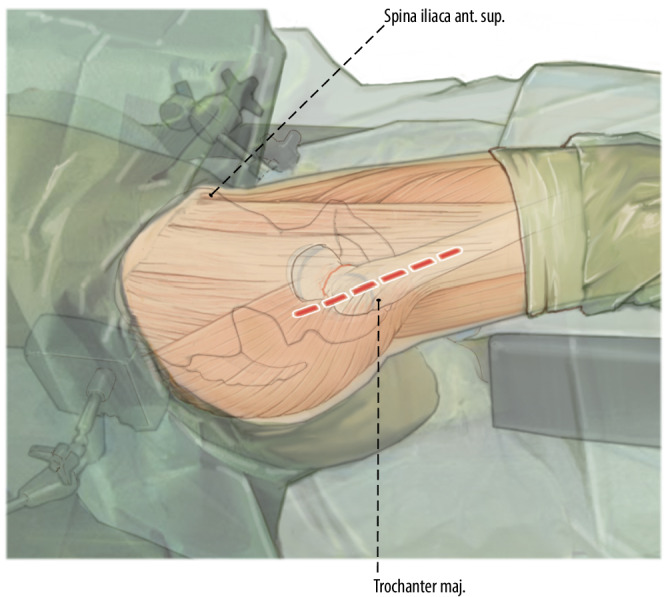

**Figure 4:**
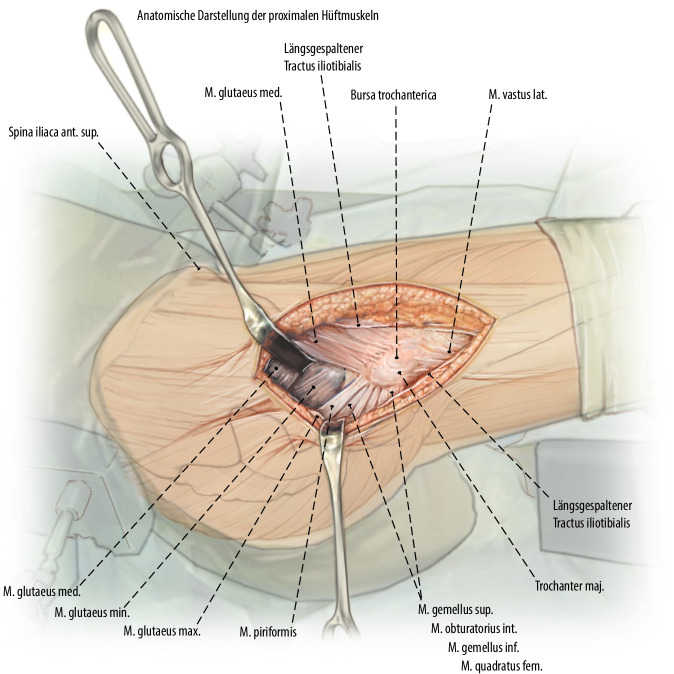

**Figure 5:**
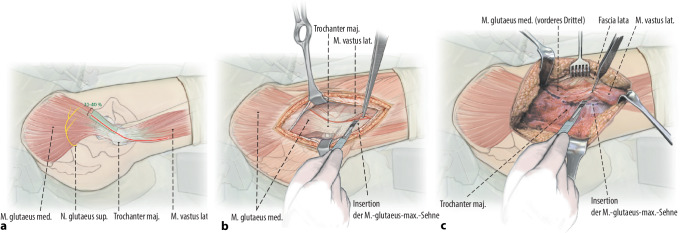

**Figure 6:**
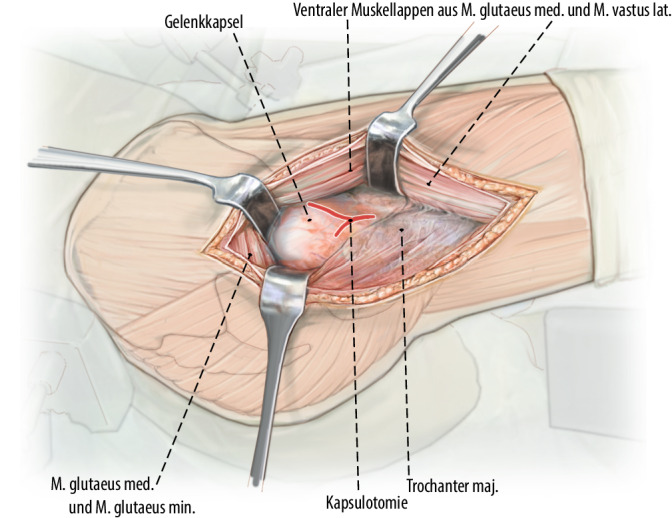

**Figure 7:**
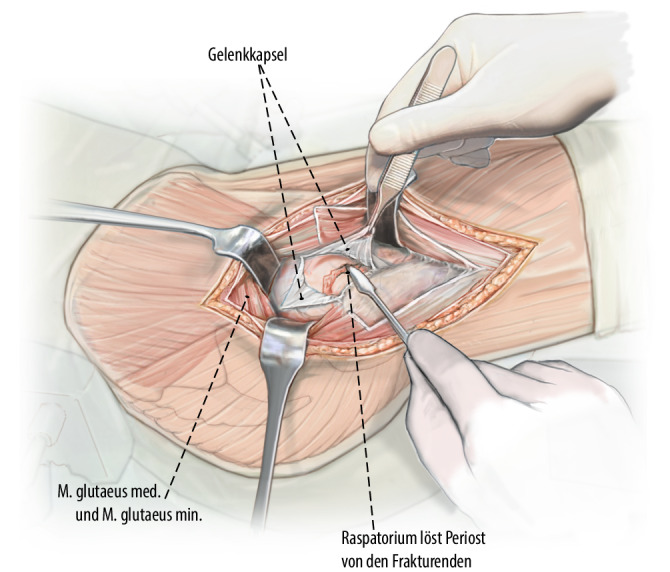

**Figure 8:**
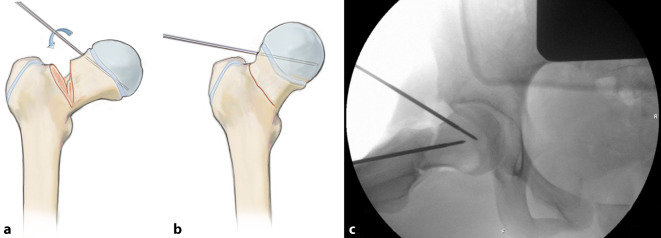

**Figure 9:**
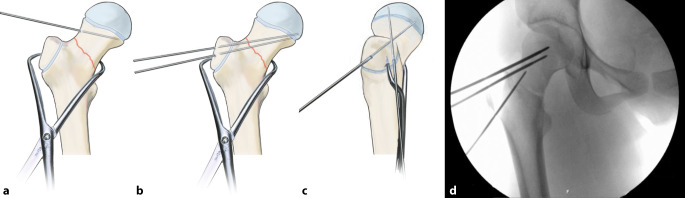

**Figure 10:**
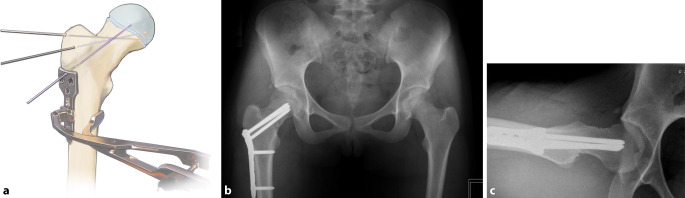

**Figure 11:**
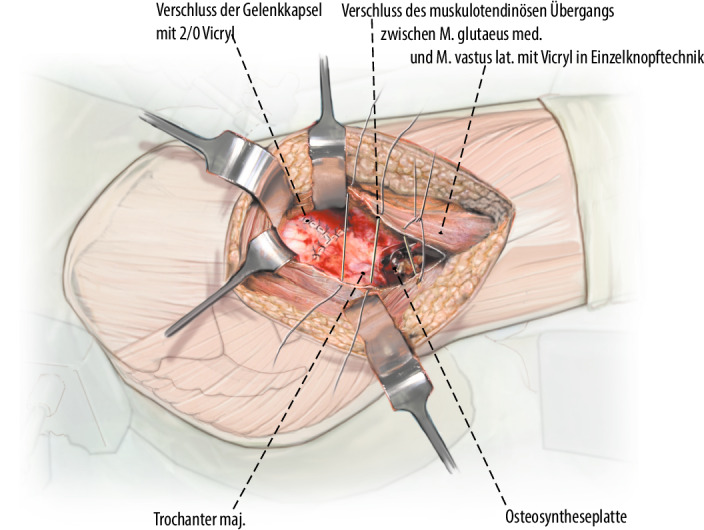

**Figure 12:**
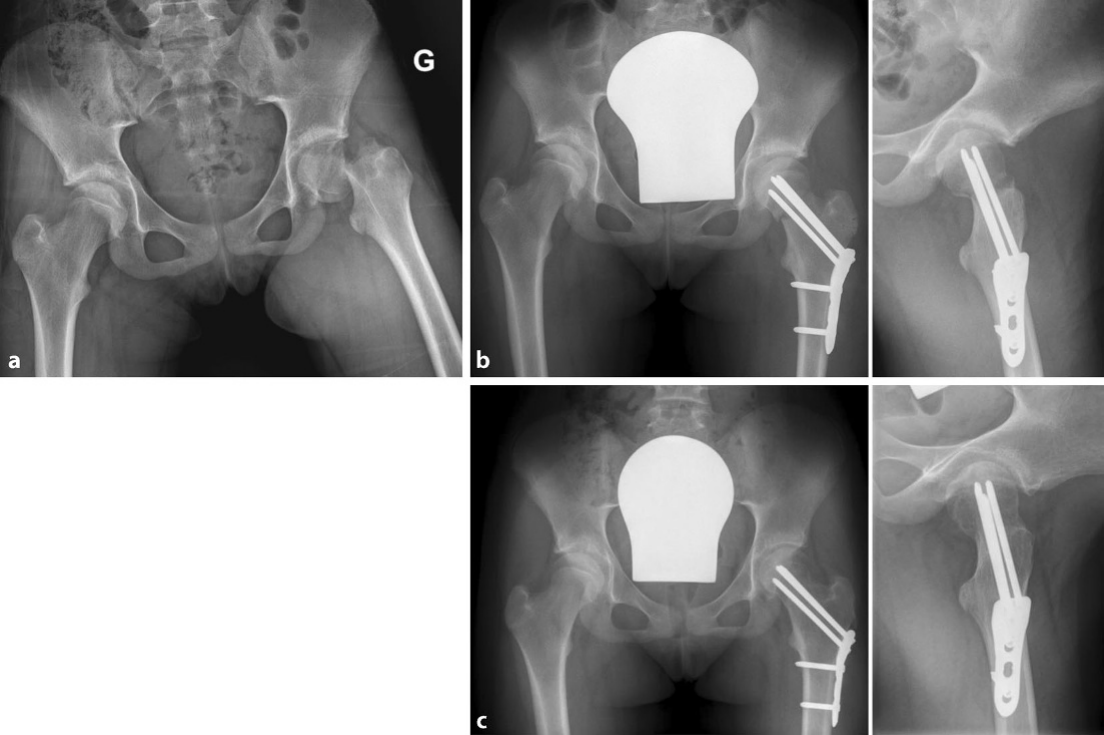

## Postoperative Behandlung

- Teilbelastung an Gehstöcken „touch down“ für 4 bis 6 Wochen (je nach Alter des Patienten)
- Bei Bedarf Motorschiene zur Verbesserung der passiven Mobilisation
- Flexion bis 90° erlaubt
- Keine aktive Abduktion und passive Adduktion (Schutz des M. glutaeus medius)
- 4 bis 6 Wochen postoperativ Konsolidationsröntgen und bei ausreichender Konsolidation Belastungsaufbau innerhalb 7 bis 14 Tagen
- Radiologische Kontrolle nach 3 Monaten, dann bei gutem Verlauf Sportfreigabe
- Langzeitkontrollen bis mindestens 2 Jahre postoperativ sind zu empfehlen.

## Fehler, Gefahren, Komplikationen und ihre Behandlung

- Operationszeitpunkt so schnell wie möglich, bei dislozierten medialen Schenkelhalsfrakturen, um das Risiko einer avaskulären Hüftkopfnekrose (AVN) zu minimieren
- Präparation zu weit nach dorsal (Gefahr der Schädigung der retinakulären Äste der A. circumflexa medialis) mit Gefahr der AVN
- Verletzung des N. glutaeus superior bei zu proximaler Splittung des M. glutaeus medius oder zu viel Traktion mit den Hohmann-Haken, meist spontane Erholung nach 3 bis 6 Monaten
- Insuffiziente Reposition (Malunion), schnellstmögliche Revision
- Insuffiziente Stabilisation (Schmerz, sekundäre Dislokation), Revisionsosteosynthese

## Ergebnisse

In den letzten 10 Jahren wurden 29 Patienten (17 Jungen, 12 Mädchen) im Durchschnittsalter von 10 Jahren mithilfe dieses Operationszuganges operiert. Die klinischen und radiologischen Ergebnisse sind exzellent. Die Abduktorenkraft wurde nicht beeinträchtigt, und die Hüftbeweglichkeit 3 Monate postoperativ war symmetrisch. Eine Materialentfernung erfolgte im Durchschnitt nach 9 bis 12 Monaten bei vollständiger Konsolidation der Fraktur. Eine operationszugangsbedingte Revision oder Komplikation trat nicht auf. Lediglich bei 3 Patienten musste eine Revisionsoperation aufgrund einer insuffizienten Reposition (1 Patient) oder einer insuffizienten Fixation der Fraktur (2 Patienten, Platte zu kurz, Schrauben nicht winkelstabil fixiert) durchgeführt werden. Eine fraktur- und/oder operationsbedingte Hüftkopfnekrose wurde in keinem Fall beobachtet.
